# Inadvertent Acute Lipid Injectable Emulsion Overdose Resulting in Fat Overload Syndrome and Pancreatitis in a Patient with TPN Dependence

**DOI:** 10.1097/PG9.0000000000000146

**Published:** 2021-11-29

**Authors:** Michael Schlegelmilch, Joshua Feder, David Creery

**Affiliations:** From the * Department of Pediatrics, Faculty of Medicine, Children’s Hospital of Eastern Ontario, University of Ottawa; † Department of Pediatrics, Division of Critical Care, Faculty of Medicine, Children’s Hospital of Eastern Ontario, University of Ottawa.

## Abstract

We report a case of an acute, inadvertent, 7.5-fold intravenous lipid emulsion overdose with 20% SMOFlipid in an 11-month-old female with tetratricopeptide repeat domain 7A (TTC7A) mutation, intestinal failure, and parenteral nutrition dependence. The overdose resulted in critical deterioration with evidence of fever, metabolic acidosis, respiratory failure, and pancreatitis that resulted in admission to the intensive care unit. This is a unique case of fat overload syndrome with acute pancreatitis following an accidental lipid injectable emulsion overdose in a pediatric patient.

## INTRODUCTION

Fat overload syndrome results from elevated triglyceride levels that occur when intravenous lipid emulsion (ILE) infusion rates exceed the rate of hydrolysis, free fatty acid uptake, and clearance (1). The risk of fat overload syndrome exists with many ILEs, including those that contain fish oil emulsions. Symptoms and signs include respiratory distress, fever, headache/irritability, jaundice, hepatosplenomegaly, cytopenias, and coagulopathy. Treatment focuses on support of the respiratory, hepatobiliary, and hematologic complications as needed. The resultant hypertriglyceridemia resolves once the ILE infusion is stopped. Fat overload syndrome can also be associated with pancreatitis, described in the adult literature. ILE overdoses causing fat overload syndrome in infants and children have been reported in the medical literature since the 1980s; however, no cases report fat overload syndrome causing pancreatitis in pediatric patients (2,3). We present an infant with inadvertent ILE overdose causing fat overload syndrome and pancreatitis in a child.

## CASE REPORT

The patient was born at 33 weeks and 5 days following pregnancy complicated by hydrops fetalis, severe ascites, and a fetal MRI suggestive of bowel perforation. She was delivered by Cesarean section for maternal rupture of membranes following an amnioreduction. She was vigorous at birth but intubated immediately and transferred to a tertiary care pediatrics center on her first day of life. Within 15 days, she underwent three laparotomies for meconium peritonitis and ultimately had a pylorus repair, duodenojejunostomy with end ileostomy, and gastrostomy tube insertion. She also screened positive on her newborn screen for severe combined immunodeficiency (SCID), which raised suspicion a mutation in tetratricopeptide repeat domain 7A (TTC7A), an autosomal recessive mutation known to cause hereditary multiple intestinal atresia with combined immunodeficiency (4,5). SCID was confirmed by genetic testing. She was not eligible for multivisceral transplant and thus became parenteral nutrition (PN) dependent for intestinal failure.

At 11 months of age she was still in our pediatric medicine ward for PN dependence and intestinal failure-related liver disease; she had never been discharged from hospital due to complex social challenges and for need for home PN. Her PN was prescribed with a glucose infusion rate of 17.9 mg/kg/min and lipid injectable emulsion (SMOFlipid [Fresenius Kabi; Germany]), which is the ILE used at our center. She received PN and lipid injectable emulsion in a 2-1 solution. PN was prescribed to start at 18:00 each evening and run over 18 hours. ILE was prescribed at 3 g/kg/day of 20% lipid injectable emulsion, which ran at a pump rate of 5.5 mL/h. One night around midnight, she developed tachycardia, tachypnea, low urine output, irritability, fever, and respiratory distress with increased oxygen requirements. Blood pressure was normal. Venous blood gas showed a metabolic acidosis with pH of 7.22, PaCO2 of 37 mm Hg and HCO3 of 15 mmol/L. Lactate was 1.4 mmol/L, glucose and electrolytes were normal. Lipase was 333 U/L (normal 12–46 U/L) and serum triglycerides, which resulted at 12:00 pm, were 1398.23 mg/dL (<250 mg/dL). At 8:00 am nursing handover, it was discovered that the lipid infusion rate was 37 mL/h for 14 hours, which was the prescribed rate of her protein-dextrose solution. This resulted in a lipid infusion rate of 1.12 g/kg/h and a total dose of 15.68 g/kg. The upper limit of the recommended infusion rate is 0.15 g/kg/h (6). The infusion was immediately stopped.

Over 24 hours, she had progressive abdominal distension, respiratory distress, and irritability. She was transferred to the pediatric intensive care unit (PICU) and required endotracheal intubation for hypercapneic respiratory failure approximately 40 hours postoverdose. A CT scan of her abdomen showed a worsening of known hepatosplenomegaly, severe ascites and a bulky, edematous, and heterogeneous appearance of the pancreas without cyst or abscess formation. She developed mild coagulopathy with a peak INR of 1.65, and PTT of 42 seconds 12 hours post ILE overdose. Fibrinogen was normal, and while she had no spontaneous hemorrhage, she developed thrombocytopenia (nadir 66 × 10^9^/L) and anemia (hemoglobin nadir of 60 g/L) by 48 hours postoverdose.

She remained intubated and ventilated for 5 days, received vitamin K for 3 days, one transfusion of packed red blood cells and aggressive diuresis with 5% albumin and intravenous furosemide for medical management of severe ascites. A peritoneal drain was considered but not pursued given her previous surgical history and high likelihood of peritoneal adhesions. Serum triglyceride and lipase levels normalized within 24 hours of discontinuing ILE infusion, and liver enzymes remained stable during convalescence. She was extubated by day 7 post-ILE overdose and transferred from the PICU on day 14.

## DISCUSSION

Our patient’s complex hepatobiliary physiology and intestinal failure may have altered lipid metabolism and predisposed her to pancreatitis. At the time of ILE overdose, our patient had a baseline mild elevation in gamma-glutamyl transferase (275-327 U/L), although had normal bilirubin and pigmented stools. TTC7A is also highly expressed in the pancreas in both exocrine and endocrine glands including in pancreas of patients with hereditary multiple intestinal atresias (7). However, our patient had no previous episodes of pancreatitis, elevated lipase, or known reflux into her hepatobiliary ducts. Hypertriglyceridemia can cause small duct obstruction of the pancreas when triglyceride levels exceed 10 mmol/L, and chylomicrons can obstruct pancreatic capillary blood flow leading to ischemia and inflammation (8). Higher levels of free fatty acids in the pancreas can trigger the release of reactive oxygen species, inflammatory mediators, and pancreatic digestive enzymes, also leading to pancreatic inflammation and tissue damage (9).

Our patient’s thrombocytopenia was also attributable to the fat overload. Her baseline platelet levels were 250 × 10^9^/L, and she had no preexisting thrombocytopenia despite having splenomegaly. We speculate that her acquired thrombocytopenia was due to platelet phagocytosis of lipid causing platelet dysfunction, which has been reported previously (10).

The mainstay of treatment for fat overload syndrome is to support the gastrointestinal, respiratory and hematologic complications that arise. Plasma or volume exchange has been used with good effect in pediatric cases of fat overload syndrome (11,12). Our patient was considered for plasma exchanged but supportive treatment was pursued as her lipemia cleared within 18 hours of discontinuing the lipid injectable emulsion infusion, and she did not decompensate until more than 24 hours following her overdose. By the time, she required PICU admission, her serum lipids were 1.08 mmol/L. Prior reports suggest that heparin increases circulating lipoprotein lipase, hepatic lipase, and accelerates clearance of hyperlipidemia. A recent review suggests heparin may be helpful in premature infants where lipid clearance may be impaired (13).

Earlier reports of fat overload syndrome document chronic rather than acute accidental overdose of standard soybean oil emulsions, which clear from the serum very slowly (14). Other preparations of ILE, such as lipid injectable emulsion (30% soy bean oil, 30% medium chain triglycerides, 25% olive oil, and 15% fish oil) or pure fish oil emulsion (Omegaven [Fresenius Kabi; Germany]), which contains 100% fish oil, have a larger ratio of omega-3 fatty acids, are less inflammatory, have more rapid serum triglyceride clearance and are more favourable in patients with PN cholestasis, liver steatosis, and fibrosis (15). Of note, pure fish oil emulsion is FDA approved for use in pediatrics, unlike lipid injectable emulsion. The enhanced clearance rate in fish oil based ILE preparations may be protective against pancreatitis. Pediatric patients who have received accidental ILE overdoses of pure fish oil emulsion, up to 5.0 g/kg/hour, did not suffer fat overload syndrome (16).

This is one of the first reported cases of pancreatitis following acute lipid injectable emulsion overdose in a medically complex pediatric patient with PN dependence. Our case demonstrates that fat overload syndrome with pancreatitis can be associated with rapid infusions of lipid injectable emulsion and cause significant patient harm. ILEs are high alert medications and errors in swapping pump rates with the protein-dextrose solution, as was seen in our case, are reported elsewhere in the literature (17). Institutional safety policy for nursing to consistently and correctly perform medication safety checks is critical in reducing the risk of medication error from PN. In response to this serious safety event, our nursing medication check policies were amended so that safety checks were performed by two nurses, both in the medication room as well as at the bedside before administering high alert medications. Our pharmacy policy also changed so that lipid injectable emulsion is dispensed in patient specific volumes that are limited to 24 hour prescribed volumes.
